# Preservation of Raw *Crassostrea gigas* Oyster Meat: Effects of Weak Organic Acid Marination on Physicochemical, Microbiological and Sensory Properties

**DOI:** 10.17113/ftb.64.02.26.9023

**Published:** 2026-06-15

**Authors:** Fabiele Bernardi, Felipe Matarazzo Suplicy, Robson Ventura de Souza, Luiza Tiemi Morikawa Shiose, Marilia Miotto, Giustino Tribuzi

**Affiliations:** 1 Department of Food Science and Technology, Federal University of Santa Catarina, Admar Gonzaga Highway, n.1346, Florianópolis, SC 88034-000, Brazil; 2 Center for Aquaculture and Fisheries Development, Agricultural Research and Rural Extension Company of Santa Catarina, Admar Gonzaga Highway, n.1188, Florianópolis, SC 88034-000, Brazil

**Keywords:** bivalve molluscs, oyster meat, shellfish consumption, shelf life, sensory analysis, semi-preserved products

## Abstract

**Research background:**

Oysters have high nutritional value; however, their short shelf life limits their commercialisation to areas close to production sites. Affordable and accessible processing techniques that extend shelf life could expand both market reach and consumer access to oysters. This study evaluates the physicochemical, microbiological and sensory properties of raw *Crassostrea gigas* oyster meat semi-preserved with weak organic acids and saline solutions under refrigerated storage (4 °C).

**Experimental approach:**

Aliquots of (100±2) g of raw oyster meat were placed into plastic containers containing different solutions: sterile deionised water only (negative control treatment, labelled W), base solution (NaCl, 5 % *m*/*V*) only, base solution with 2 % citric acid (CA), base solution with 2 % lactic acid (LA),and base solution with 2 % acetic acid (AA). Sensory, physicochemical and microbiological characteristics of the semi-preserves were monitored for 16 days.

**Results and conclusions:**

Incorporating weak acids into the marination solutions effectively inhibited the growth of mesophilic and psychrotrophic bacteria in semi-preserved oysters during 16 days of refrigerated storage. In contrast, water and NaCl treatments exceeded the recommended limit of 5 log CFU/g for seafood after 3 and 11 days, respectively. By day 16, total volatile basic nitrogen (TVB-N) values indicated early spoilage in water ((30.0±1.1) mg/100 g), satisfactory freshness in NaCl ((24.5±2.6) mg/100 g) and CA ((18.3±1.1) mg/100 g), and excellent freshness in LA ((14.7±0.0) mg/100 g) and AA ((15.0±1.8) mg/100 g). Thiobarbituric acid reactive substances (TBARS) assay values, expressed as malondialdehyde (MDA), remained below 3 mg/kg in all treatments, indicating good oxidative stability. Among the acids, AA maintained higher pH values (3.78 on day 16) than CA (3.26) and LA (3.14) and showed the lowest microbial loads; however, it received the highest scores for acid odour (median=5.35) and the lowest for characteristic oyster odour. CA and LA produced sensory profiles more similar to fresh oysters, with higher characteristic odour scores and lower acid odour scores, but slightly higher spoiled odour scores (still low in absolute terms). Overall, AA was the most effective for microbiological and physicochemical preservation, while CA and LA offered better sensory acceptance. These results highlight the potential of weak organic acids, particularly AA, as a low-cost method to extend the shelf life of raw oysters to at least 16 days under refrigeration.

**Novelty and scientific contribution:**

This study evaluates the effects of marination with weak acids on the physicochemical, microbiological, and sensory properties of raw oyster meat. The wide range of parameters analysed highlights not only its suitability for consumption but also consumer preferences based on sensory aspects such as colour and odour. The combined findings can assist the industry in selecting the most appropriate acid for developing different oyster-based products.

## INTRODUCTION

Oysters are marine bivalves of high economic and nutritional value, and the most cultivated shellfish worldwide. The global aquaculture market was valued at USD 9251.5 million in 2024 and is projected to reach USD 11 770.5 million by 2031, growing at 3.5 % annually (*1*). The Pacific oyster (*Crassostrea gigas*), the most widely consumed species, dominates global production, with China contributing nearly 89 % of the total (*2*). In addition to their market relevance, oysters are a rich source of high-quality protein, essential amino acids, and trace minerals (*3*).

However, oysters are highly perishable due to high water activity, active endogenous enzymes, and susceptibility to microbial contamination (*4*). Maintaining quality and safety during storage and distribution is challenging (*3*), and the raw consumption of live oysters poses potential health risks associated with pathogenic contamination (*5*). Preservation strategies include refrigeration, freezing, high-pressure processing (HPP), modified atmosphere packaging (MAP), and natural preservatives (*3*-*16*).

Marination is a traditional technique that uses natural products, such as weak organic acids, as a mild preservation method. In addition to enhancing flavour, marination can inhibit microbial growth through the combined effects of organic acids and other natural preservatives (*10*, *12*-*14*, *17*). Marinated seafood products are valued for their convenience, sensory appeal, and extended refrigerated shelf life (*14*). Organic acids such as acetic, citric, and lactic acids exhibit antimicrobial activity by penetrating microbial membranes in undissociated form, acidifying the cytoplasm, and disrupting metabolism. They also destabilise membranes, impair nutrient transport, and slow enzymatic and oxidative reactions, helping preserve colour, texture, and flavour (*13*, *14*, *16*).

Demand for ready-to-eat oysters (*4*) highlight the need for preservation strategies that ensure safety and quality while meeting consumer expectations. Semi-preserves are products that are not subjected to pasteurisation and therefore remain essentially in their raw state. As such, they represent an attractive option for consumers seeking minimally processed products. However, few studies have investigated the production of semi-preserves from raw, shucked oysters, highlighting a gap in the development of minimally processed oyster products. The present study evaluates the effect of marination with weak organic acids on the quality and shelf life of refrigerated shucked oysters, aiming to support the development of safe, nutritious, and convenient oyster products.

## MATERIALS AND METHODS

### Sample and solution preparation

Approximately 480 Pacific oysters (*Crassostrea gigas*) were harvested in November 2022 from a marine farm located in the southern part of Santa Catarina Island, Brazil (27°49′5.10″S; 48°34′4.84″W), and mechanically washed. The oysters were transported to the Fish Technology Laboratory at the Federal University of Santa Catarina (UFSC), where they were manually shucked and rinsed with deionised water.

For the preparation of marination solutions, food-grade acids were diluted in a base solution consisting of deionised water and NaCl (5 % *m*/*V*). The tested solutions, their abbreviations, and compositions were as follows: W=sterile deionised water only (negative control), NaCl=base solution only (Diana Salt, Paranaguá, Brazil), CA=base solution with 2 % (*m*/*V*) citric acid (Allimentari, São Paulo, Brazil), LA=base solution with 2 % (*V*/*V*) lactic acid (Allimentari), and AA=base solution with 2 % (*V*/*V*) acetic acid (Heinig, Brusque, Brazil). The composition of the marination solutions were selected according to Tribuzi *et al.* (*18*). All solutions were prepared one day prior to the production of the semi-preserves and stored under refrigeration at 4 °C until use.

### Preparation of semi-preserves

Aliquots of (100±2) g of raw oyster meat were placed in plastic containers (300 mL; Altacoppo, Carapicuíba, Brazil), and the containers were organised into five treatment groups (12 containers per group). Each container was filled with 200 g of the corresponding marinade solution (meat to marinade mass ratio of 1:2) and hermetically sealed. The semi-preserves were stored at (4±1) °C in a biochemical oxygen demand (BOD) incubator (TE371/240 L; Tecnal, Piracicaba, Brazil) for 16 days. Microbiological, physicochemical, and sensory analyses were conducted after 2, 7, 11 and 16 days of storage according to Puértolas *et al.* (*9*). At each sampling point, three containers per treatment were withdrawn from the BOD incubator and transferred to the laboratory for analysis.

### Characterisation of raw oyster meat

Ash content was determined by weighing the residue after incineration at (550±10) °C for 5 h, as described by Instituto Adolfo Lutz (*19*). Protein content was determined using the Kjeldahl method (*20*). Crude fat content was determined by extracting the sample with petroleum ether using a Soxhlet apparatus (*21*). Total carbohydrate content and caloric value (*E*/kJ) were calculated by difference, according to the following equations (*22*):


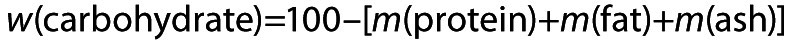
 /1/


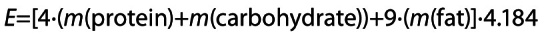
 /2/

### Analytical determinations

Samples were removed from the BOD incubator at each experimental time and kept at room temperature for 30 min, then transferred to sieves for 2 min to allow separation of the liquid from the meat. The oyster meat and the drained liquid were weighed separately, and the mass loss (in %) was calculated according to Liu and Zhang (*23*), using the following equation:


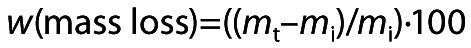
 /3/

where *m*_i_ is the oyster meat initial mass and *m*_t_ is the mass at time *t* during the marinating process.

The amount of undissolved matter (salt, acid, and oyster solids) in the solutions was determined. Approximately 3 g of the liquid drained from the semi-preserved samples was weighed and placed in an oven at 105 °C for 24 h, following the procedure proposed by Instituto Adolfo Lutz (*19*). The marinade liquid was centrifuged (Kasvi, Pinhais, Brazil) for 20 min at 2100×*g*, and the turbidity of the supernatant was measured using a turbidimeter (DM-TU; Digimed, São Paulo, Brazil).

The samples were homogenised, and their pH was measured with a benchtop pH meter (Edge®; Hanna Instruments, Woonsocket, RI, USA) by inserting the electrode directly into each sample. Moisture content was determined by the gravimetric method (*19*), by weighing thawed samples (2 to 10 g) of oyster before and after drying in an oven (Biopar, São Paulo, Brazil) at 105 °C until reaching a constant mass.

The total volatile basic nitrogen (TVB-N) was analysed according to the Brazilian official methods for foods of animal origin (*24*), with some modifications. A mass of 5 g of oyster meat was homogenized with 45 mL of 6 % perchloric acid solution (Neon, São Paulo, Brazil) using a Turrax homogenizer (Ultra-Turrax T18; IKA, Staufen, Germany) at 12 000 rpm for 2 min and filtered through a filter paper. A 25-mL aliquot of the filtrate was placed in the steam distillation apparatus (Tecnal) along with five drops of phenolphthalein (Synth, Diadema, São Paulo) and 3.25 mL of 20 % sodium hydroxide (Synth). The distillate was collected in an Erlenmeyer flask with 50 mL of 3 % boric acid (Neon) and five drops of Tashiro pH indicator. Distillation was considered complete when a final volume of 100 mL was obtained (50 mL of distillate and 50 mL of solution), and then the solution was titrated with 0.1 M hydrochloric acid (Neon). Finally, a blank test was performed, replacing the 25-mL filtrate with 25 mL of 6 % perchloric acid solution. The results were expressed in mg of TVB-N per 100 g of oyster meat.

Lipid oxidation was assessed by evaluating TBARS as described by Vyncke (*25*) and adapted by Fogaça *et al.* (*26*). A mass of 10 g of the oyster sample was homogenised with 7.5 % trichloroacetic acid (TCA) (Dinâmica, Indaiatuba, Brazil) for 5 min. The homogenate was filtered through Whatman no. 1 filter paper. The supernatant (5 mL) was mixed with 5 mL of 0.02 M thiobarbituric acid (TBA) solution (Sigma-Aldrich, Merck, St. Louis, MO, USA). The sample was heated at 95 °C for 10 min and cooled on ice or at room temperature for 10 min. Absorbance was measured at 532 nm using a UV-Vis spectrophotometer (model K37-UVVIS; Kasvi). 1,1,3,3-Tetraethoxypropane (TEP; Sigma-Aldrich, Merck, Dublin, Ireland) was used as a standard. The TBARS value was expressed as mg malondialdehyde per kg (mg/kg).

To determine the NaCl concentration, 0.5 g of the sample was homogenized in distilled water using an Ultra-Turrax (Ultra-Turrax T18; IKA) at 12 000 rpm for 2 min. The solution was then brought up to 40 mL with distilled water and centrifuged at 2100×*g* for 10 min (K14-4000; Kasvi). A 500 μL aliquot of the supernatant was analysed using a chloride analyser (Chloride Analyzer M926S; Cole-Parmer, Cambridge, UK). All analytical determinations were performed in triplicate.

### Evaluation of the mass transfer

The water gain (WG) and salt gain (SG) were determined in g/100 g using the following equations, respectively, which allowed calculating the mass transfer between the oysters and the tested solutions (*18*):


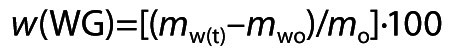
 /4/


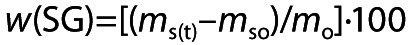
 /5/

where *m*_o_ is the initial mass of the oyster meat, *m*_w(t)_ is the water content at time *t*, *m*_wo_ is the initial water content in oyster meat, *m*_s(t)_ is the salt content at time *t*, and *m*_so_ is the initial salt content in oyster meat.

### Microbiological analysis

Sterile bags were filled with 10 g of oyster meat and 90 mL of 0.1 % peptone water with 0.05 % sodium chloride (Merck, Pinhais, Brazil), and the samples were homogenised. The mesophilic bacteria were counted following the ISO 4833-1:2013/Amd 1 method (*27*). Serial decimal dilutions were inoculated using the pour plate technique in sterile Petri dishes, followed by the addition of previously melted and cooled plate count agar (PCA; Merck). The plates were incubated at (30±1) °C for (48±2) h, and the results were expressed as CFU/g. Psychrotrophic bacteria were counted according to the American Public Health Association method (*28*). Serial decimal dilutions were inoculated by surface spreading on plate count agar (PCA). Plates were incubated at (7±1) °C for 10 days, and the results were expressed as CFU/g.

### Sensory analysis

#### Colour parameters

A computer vision system (*29*) was used to determine the colour parameters L*, a* and b* of the CIELAB scale for the samples, with L* representing lightness (black to white), a* representing red/green and b* representing yellow/blue. Images of the samples were captured with a camera (Canon EOS1100D; Nikon Corporation, Taiwan, Japan) and processed using ImageJ v. 1.6.0 software (*30*). A plug-in (colour space converter) converted the colour from the red, green and blue (RGB) system to the CIELAB scale, and the total colour difference (ΔE*) was calculated according to the following equation:


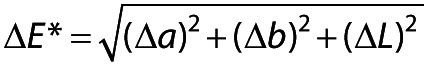
 /6/

The analyses were performed in triplicate. Colour analysis was not performed on day 11 due to an insufficient amount of oyster meat, which did not meet the minimum requirements established by the analytical protocol.

#### Odour parameters

The sensory analysis of marinated raw oyster meat (approved by the UFSC Ethics Committee, process number 58147722.5.0000.0121) was organised into four stages: (*i*) recruitment, (*ii*) training, (*iii*) selection, and (*iv*) sensory analysis. E-mails were sent to students on food science and aquaculture courses to recruit volunteer evaluators. A Google Forms questionnaire was then used to assess their eating habits, availability, and health status. Individuals were selected if they were regular consumers of oysters and denied any health problems (allergy, hypertension, diabetes, rhinitis). The methodology proposed by Meilgaard *et al*. (*31*) was used to train the volunteers. Colour and odour scales were employed, adapting the NBR ISO 8586:2016 (*32*) approach. Evaluators were asked to arrange randomly organised samples in ascending order of colour and odour according to NBR ISO 8587:2015 (*33*). They were also asked whether they would consume the evaluated product. For the evaluator selection process, the triangular test, following the NBR ISO 4120:2013 (*34*) standard, along with an unstructured scale, was used. Only evaluators who achieved a 75 % accuracy rate in the tests were selected for the study. Sensory evaluation of the oyster samples was performed by a panel of 10 evaluators. The consumer group included 50 % males and 50 % females. The age ranges were: 20–25 years (20 %), 26–35 years (40 %), 36–45 years (30 %) and 46–55 years (10 %). For the sensory analysis, unstructured 10 cm scales (*35*) were used. Fresh oyster meat was provided to the evaluators as a reference (methodology proposed by Silva *et al.* (*36*), with adaptations), and they were asked to assess the intensity of the odour (characteristic, acidic, spoiled, and putrid) of the treated oysters. The closer the reported value was to zero, the lower the odour intensity perceived by the evaluators; the closer to ten, the higher the intensity, with the extremes being ’none’ and ’extremely strong’.

### Statistical analysis

The data on pH, total volatile bases, lipid oxidation, colour, and mesophilic bacteria counts, both in the tested solutions and in the meat, met the assumption of normality according to the Shapiro-Wilk test. Therefore, two-way analysis of variance (ANOVA) followed by Tukey's test (95 %) as a *post-hoc* analysis was used to investigate the effects of treatments and storage time on these parameters. The data on turbidity, insoluble solids content, psychrotrophic bacteria counts, and sensory evaluation deviated from normality according to the Shapiro-Wilk test; for these, the Kruskal-Wallis test followed by pairwise comparisons (Dunn’s test) was used. All statistical analyses were performed using R software v. 4.3.1 (*37*).

## RESULTS AND DISCUSSION

### Proximate composition of raw oyster meat prior to marination

The moisture, ash, lipid, protein, carbohydrate, and caloric values of fresh oyster meat used in this study are summarised in Table 1. Several factors, such as season, environmental conditions and cultivation depth, influence the proximate composition of *Crassostrea gigas* (*38*). In this study, the moisture content of oyster meat was (82.5±0.1) g/100 g, within typical ranges for various oyster species (*39*). The protein mass fraction of (9.4±0.0) g/100 g aligns with the lower ranges often reported for *C. gigas* and related species, commonly between 9 and 14 g/100 g depending on environmental and reproductive parameters (*39*). The lipid mass fraction of (1.7±0.1) g/100 g is lower than usually observed in *C. gigas* ((2.5–4.0) g/100 g), yet within the variability influenced by season and gonadal maturation (*40*). Ash mass fraction of (1.2±0.0) g/100 g falls slightly below the expected values of (2.0–3.5) g/100 g, possibly due to lower mineral accumulation or dilution by high moisture (*39*). Carbohydrates estimated by difference at (5.3±0.1) g/100 g reflect typical glycogen reserves outside reproductive periods (*39*). The caloric value of (308.7±3.1) kJ/100 g is consistent with the expected energy content for fresh oyster meat and highlights its profile as a low-calorie, high-quality protein source (*38*).

**Table 1:** Proximate composition and caloric value of raw oyster meat used in this study

Parameter	*w*/(g/100 g)
Moisture	82.5±0.1
Ash	1.2±0.0
Lipid	1.7±0.1
Protein	9.4±0.0
Carbohydrate	5.3±0.1
*E*/(kJ/kg)	308.7±3.1

### Effect of using different weak organic acids and NaCl on mass transfer during marination

The selection of a marination solution containing 2 % organic acid and 5 % NaCl is supported by scientific evidence demonstrating its effectiveness in preserving the quality of fishery products (*16*). The positive effects are attributed to the synergistic action of the acid and salt, which together enhance microbiological stability and slow down spoilage processes. This combination has therefore proven to be a promising strategy for marinating molluscs (*18*, *41*).

**Fig. 1:**
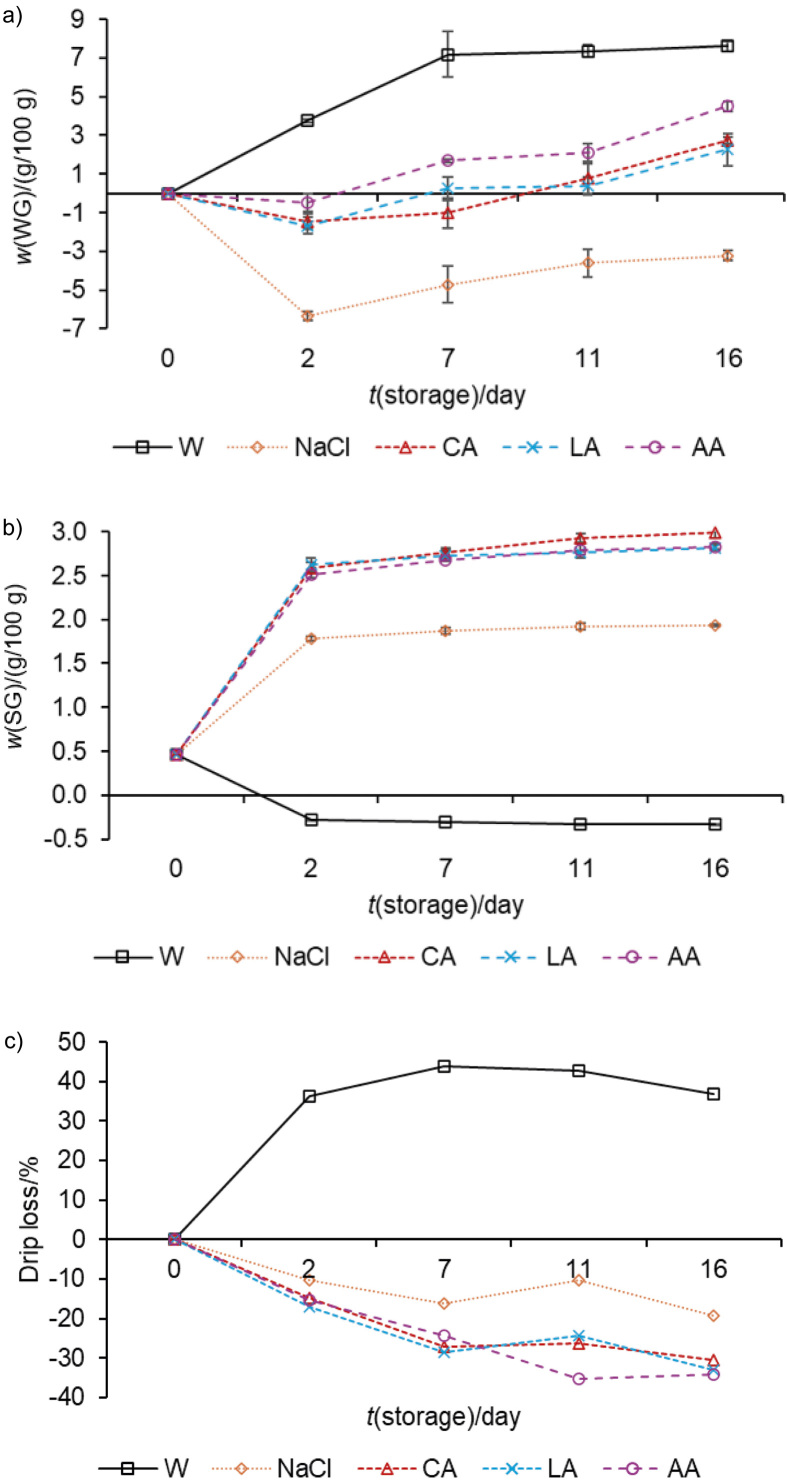
Values of: a) water gain (WG), b) salt gain (SG), and c) drip loss of the semi-preserved oyster meat during storage at 4 °C for 16 days. W=distilled water (control), NaCl=5 % sodium chloride (basic solution) and basic solution with the addition of 2 % acid: citric (CA), lactic (LA) and acetic (AA)

Fig. 1 shows the values of water gain (WG), salt gain (SG), and drip loss for semi-preserved oysters subjected to different treatments during storage at 4 °C. Water gain was significantly affected by both treatment and storage time (p<0.05), with distinct patterns observed among the treatments (Fig. 1a). Throughout the 16-day storage, the solution containing 5 % NaCl resulted in negative water gain, indicating water loss. Oysters treated with citric acid (CA) lost water until the seventh day, after which water gain was observed. For lactic acid (LA) and acetic acid (AA) treatments, water content was slightly reduced during the first two days of storage, followed by a subsequent increase.

According to Logrén *et al.* (*16*), the simultaneous presence of salt and acid reduces the pH of minimum water retention (typically corresponding to the isoelectric point) by approximately one unit, reaching around 4.5. Consequently, the dehydrating effect in the presence of NaCl is more pronounced at pH values around 4–5, due to the reduction in positive repulsion forces (R–NH_3_^+^) caused by the strong affinity of Cl^−^ anions for proteins.

A significantly higher water gain was observed for AA than for CA and LA, which may be related to protein denaturation promoted by the higher p*K*_a_ value of AA (logarithm of the acid dissociation constant: p*K*_a_(AA)=4.74; p*K*_a_(CA)=3.14; p*K*_a_(LA)=3.86) (*41*). Logrén *et al*. (*16*) reported that AA tends to counterbalance the impact of high NaCl mass fraction, thus mitigating salt-induced water loss. In contrast, CA and LA, characterised by lower p*K*_a_ values, exhibit reduced water retention capacity due to degradation of connective tissue proteins, which outweighs the swelling effect of myofibrillar proteins, as reported by Bampi *et al*. (*42*).

Both treatments and storage time significantly affected (p<0.05) salt gain (Fig. 1b). The NaCl and acid treatments resulted in salt uptake, while the control lost salt. The initial NaCl mass fraction in raw oyster meat was (0.5±0.0) g/100 g, and an increased salt gain was observed during the first two days of marination. This can be attributed to the high chemical potential gradient between the solution and the oyster meat, with salt gain tending to stabilise in the following days until equilibrium was reached (*18*).

Oyster meat from the control group gained mass until the seventh day, likely due to water uptake (Fig. 1c). In contrast, oyster meat from the other treatments lost mass until the second day and then tended to stabilise. This behaviour can be ascribed to osmotic equilibrium between the meat and the marinating medium (*18*). The mass loss observed in the NaCl and acid treatments may be linked to myosin denaturation, leading to a reduced capacity for liquid retention (*16*).

Influence of weak organic acids and NaCl on turbidity, insoluble solids content, pH, total volatile basic nitrogen, and lipid oxidation

**Fig. 2:**
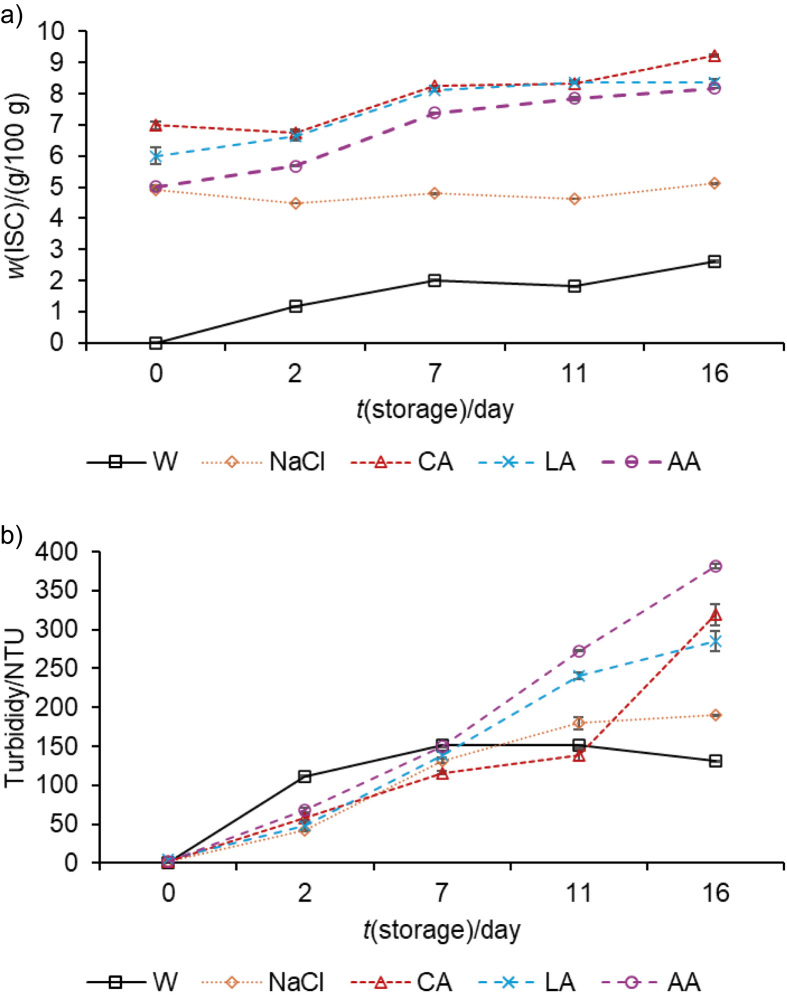
Values of: a) insoluble solid content (ISC), and b) turbidity of the solution drained from the semi-preserved oysters during storage at 4 °C for 16 days. W=distilled water (control), NaCl=5 % sodium chloride (basic solution) and basic solution with the addition of 2 % acid: citric (CA), lactic (LA) and acetic (AA)

The insoluble solid content (ISC) was significantly influenced by both storage time and treatment (p<0.05). Its values in the marinating solution (Fig. 2a) increased in all treatments over time compared with the initial values. Despite the different compositions of each treatment, no statistical differences (p>0.05) were observed among treatments on day 0. Treatments W and NaCl did not differ statistically (p>0.05) from each other throughout the 16 days of storage. The CA treatment exhibited the highest ISC values and differed significantly (p<0.05) from the other treatments from day 2 onwards. Treatments LA and AA differed significantly on days 2 and 16, while no differences were observed on days 7 and 11.

A significant interaction between storage time and treatment was observed (p<0.05) for the turbidity of the solutions. As shown in Fig. 2b, turbidity generally increased over time in all treatments except the control (water). Lower turbidity values were recorded for water and NaCl treatments than for the acid treatments. After 2 days of refrigerated storage, the NaCl treatment exhibited the lowest turbidity value, which did not differ statistically from CA, LA and AA, but differed from water. After 7 days, the CA treatment had higher turbidity than the control (water), but did not differ from the other treatments. After 11 and 16 days of storage, the AA treatment showed the highest turbidity values, differing significantly (p<0.05) from all other treatments.

**Fig. 3:**
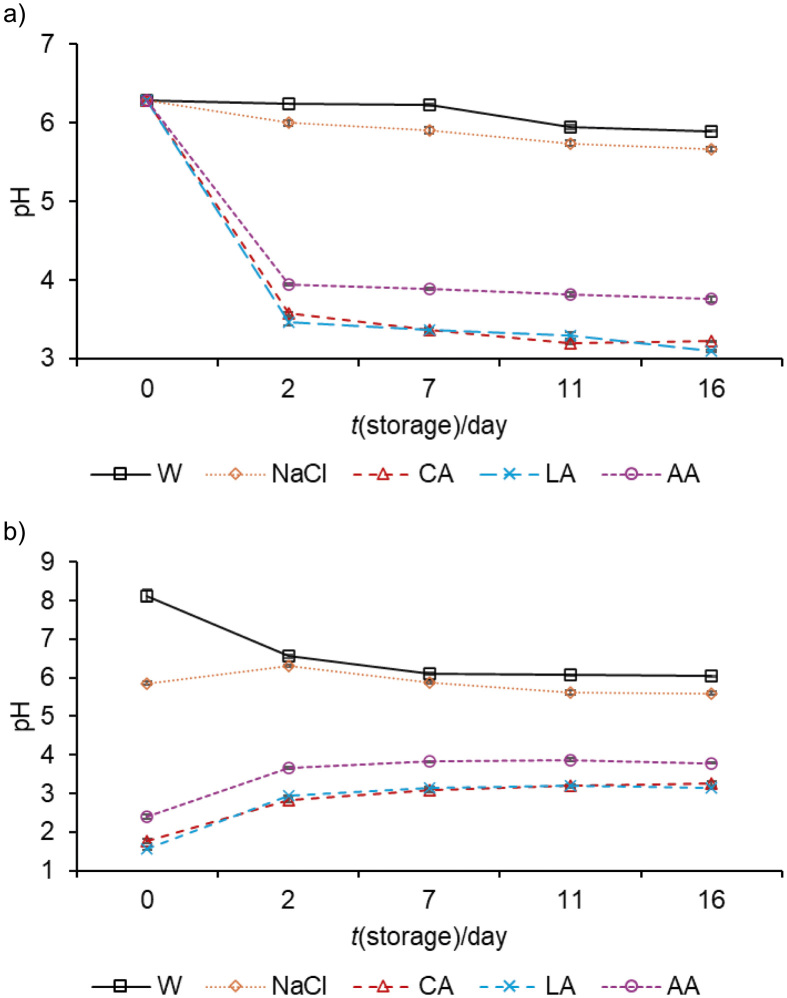
Values of: a) pH of oyster meat, and b) pH of the marinating solution of the semi-preserved oysters during storage at 4 °C for 16 days. W=distilled water (control), NaCl=5 % sodium chloride (basic solution) and basic solution with the addition of 2 % acid: citric (CA), lactic (LA) and acetic (AA)

Statistical analysis showed that pH, total volatile basic nitrogen (TVB-N), and lipid oxidation (TBARS) parameters were significantly affected by the treatments, either directly or through interaction with storage time (p<0.05). The pH of raw oyster meat was 6.3±0.0, a value comparable to that reported by Min *et al*. (*8*) for *Crassostrea gigas* (pH=6.5). After 16 days of storage, the water and NaCl treatments exhibited pH reductions of 6.4 and 9.9 %, respectively (Fig. 3a). Despite the absence of acids in these treatments, the observed pH decrease may be attributed to the fermentation of oyster glycogen reserves. The breakdown of glycogen through glycolysis into pyruvic and lactic acids results in a decrease in pH (*6*). Treatments containing acids showed a marked pH reduction, particularly during the first two days of storage. By the end of storage, the CA treatment exhibited a 48.8 % reduction, LA showed 50.9 % and AA showed a 40.4 % reduction in pH compared with the pH of fresh oyster meat. Throughout storage, the pH of oyster meat remained above pH=3 in all treatments.

The trend observed for oyster meat pH in the acid treatments was consistent with that of the marinade (Fig. 3b), showing a marked increase during the first two days of storage, followed by more stable values thereafter. The initial pH values for the CA, LA and AA solutions were 1.8±0.1, 1.6±0.0 and 2.4±0.1, respectively, and increased to 3.3±0.1, 3.1±0.0 and 3.8±0.0 by the end of the assay. The water treatment started with a pH of 8.1±0.2 and the NaCl treatment with 5.8±0.0, decreasing to 6.0±0.0 and 5.6±0.0, respectively, by the end of the assay.

The contact between the marinating solution and the oyster meat promotes the diffusion of acid and salt into the meat tissue until concentration equilibrium is reached (*12*). Silva *et al*. (*10*) reported an initial pH=4.52 and a final pH=5.17 after 35 days for cooked *Crassostrea gasar* marinated in a 2.5 % AA solution and stored at 4 °C. In a study with sardines, Dericioglu *et al*. (*43*) reported an initial pH=6.36 and a final pH=2.86 for samples marinated in 2.5 % AA and stored for three months at 4 °C.

**Fig. 4:**
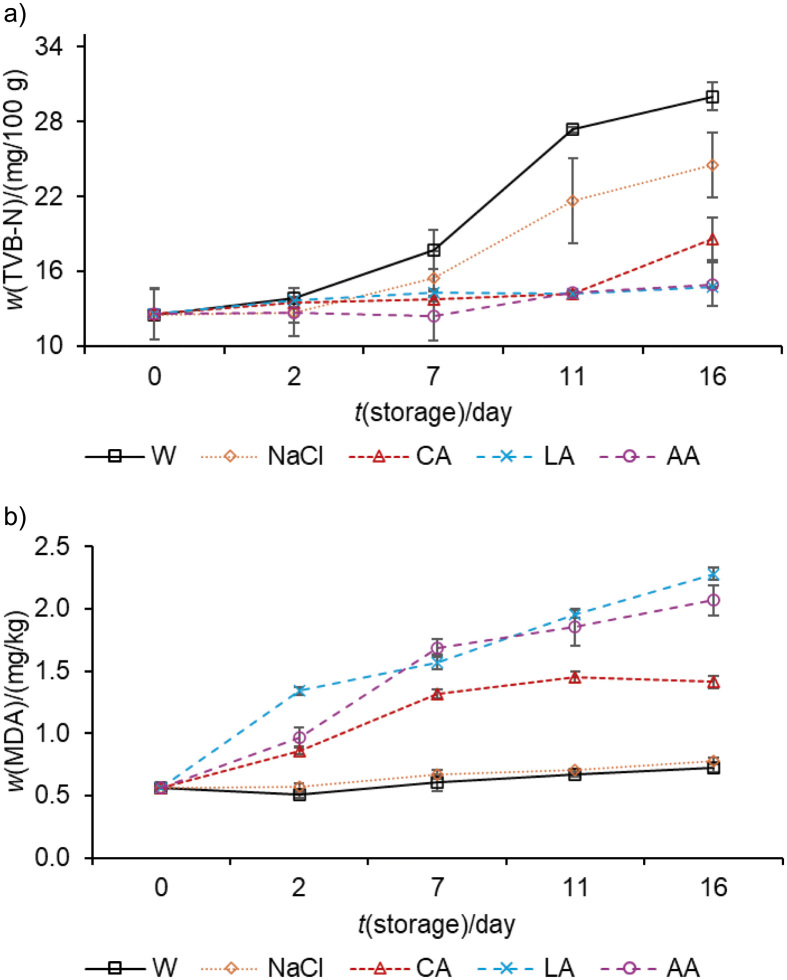
Values of: a) total volatile basic nitrogen (TVB-N), and b) lipid oxidation (TBARS as *w*(MDA)) of the semi-preserved oyster meat during storage at 4 °C for 16 days. TBARS=thiobarbituric acid reactive substances, MDA=malondialdehyde, W=distilled water (control), NaCl=5 % sodium chloride (basic solution) and basic solution with the addition of 2 % acid: citric (CA), lactic (LA) and acetic (AA)

According to Kim *et al.* (*44*), an excellent degree of freshness is associated with TVB-N values ranging from 5 to 15 mg/100 g, satisfactory freshness with values from 16 to 29 mg/100 g, the onset of spoilage with values from 30 to 40 mg/100 g, and values above 50 mg/100 g indicate severely deteriorated products. The TVB-N results obtained in this study showed an increasing trend over time (Fig. 4a), although different treatments exhibited different TVB-N values. By the end of the assay (16 days of storage), oyster meat from the water treatment was at the onset of deterioration (30.0±1.1) mg/100 g, while NaCl and CA treatments maintained satisfactory freshness (24.5±2.6) and (18.3±1.1) mg/100 g, respectively. The LA and AA treatments still exhibited excellent freshness (14.7±0.0) mg/100 g and (15.0±1.8) mg/100 g, respectively.

Dericioglu *et al.* (*43*) evaluated TVB-N values in sardine marinades and reported that they increased over time. At a 2.5 % acid, the initial TVB-N value was (6.74±0.02) mg/100 g, increasing to (8.10±0.45) mg/100 g after one month and to (15.25±0.28) mg/100 g after three months. Similarly, Silva *et al*. (*10*) reported an increase in TVB-N values for pasteurised canned oysters (*Crassostrea gasar*) during 35 days of storage, from 9.78 mg/100 g on day 0 to 11.98 mg/100 g on day 35.

Products are categorised according to TBARS values, expressed as MDA, with values below 3 mg/kg indicating excellent quality and those below 5 mg/kg indicating good quality (*43*). As shown in Fig. 4b, TBARS values increased over time; however, all treatments remained below 3 mg/kg. A similar trend has been reported in other studies. For example, in marinated sardines (*43*), the TBARS value on day 0 was (2.13±0.04) μmol/kg, increasing to (4.82±0.57) μmol/kg after three months in the group treated with 2.5 % AA.

Logrén *et al*. (*16*) investigated marination with AA, CA and LA in Baltic herring fillets and found the lowest TBARS values in fillets marinated in CA and the highest in those marinated in AA. However, MDA values increased significantly in all acid treatments when the storage period was extended to four months, reaching approx. 4–8 mg/kg. Min *et al*. (*8*) studied fresh *Crassostrea gigas* and reported a significant increase in TBARS from 0.24 mg/100 g on day 0 to 0.36 mg/100 g by day 8.

### Effect of the use of weak organic acids and NaCl in microbiological analyses

Statistical analysis showed that bacterial counts were significantly influenced by the interaction between storage time and treatment (p<0.05). The mesophilic and psychrotrophic counts are shown in Table 2. Considering 5 log CFU/g of mesophiles or psychrotrophs as the rejection limit for seafood (*45*), the water treatment had a shelf life of 3 days, while the NaCl treatment had a shelf life of 11 days.

**Table 2:** The mesophilic and psychrotrophic counts of processed oysters during shelf-life study

*t*(storage)/day	*N*(mesophilic bacteria)/(CFU/g)					*N*(psychrotrophic bacteria)/(CFU/g)				
	Treatment					Treatment				
	W	NaCl	CA	LA	AA	W	NaCl	CA	LA	AA
0	(3.4±3.3)^A^	(3.4±3.3)^A^	(3.4±3.3)^A^	(3.4±3.3)^A^	(3.4±3.3)^A^	(2.8±3.0)^bAB^	(2.9±3.0)^bAB^	(2.9±3.0)^aAB^	(2.9±3.0)^aAB^	(2.9±3.0)^aAB^
2	(3.9±2.2)^aA^	(3.5±2.8)^ab^	(3.4±3.0)^abcA^	(3.1±2.6)^bcA^	(2.8±2.2)^cA^	(1.8±1.8)^bA^	(3.0±2.7)^bA^	<2^aA^	<2^aA^	<2^aA^
7	(5.3±5.2)^aB^	(4.1±3.9)^b^	(1.8±1.8)^cdB^	(2.0±1.6)^cdBC^	(1.1±1.4)^dB^	(4.5±3.9)^bAB^	(4.6±4.4)^bAB^	<2^aAB^	<2^aAB^	<2^aAB^
11	(6.4±5.6)^aC^	(3.5±2.3)^b^	<1^cB^	(1.7±1.6)^cB^	(1.43±0.8)^cB^	(6.3±6.2)^baBC^	(4.5±4.0)^abBC^	(2.8±2.7)^aBC^	(3.0±2.7)^aBC^	(3.0±2.1)^abBC^
16	(7.8±7.3)^aD^	(3.8±3.6)^b^	(2.0±2.0)^cdB^	(2.4±2.2)^cC^	(1.2±1.5)^dB^	(6.4±5.7)^bAB^	(5.3±4.7)^bABC^	<2^aAB^	<2^aAB^	<2^aAB^

Different lowercase letters in superscript within the same row indicate significant differences (p<0.05) among treatments. Different uppercase letters in superscript within the same column indicate significant differences (p<0.05) among storage periods. W=distilled water (control), NaCl=5 % sodium chloride (basic solution) and basic solution with the addition of 2 % acid: citric (CA), lactic (LA) and acetic (AA)

The antimicrobial activity of organic acids is related to their p*K*_a_ value: the higher the p*K*_a_, the stronger the effect (p*K*_a__AA=4.74, p*K*_a__CA=3.14 and p*K*_a__LA=3.86) (*16*). Inside the cell cytoplasm, the high pH of the medium facilitates acid dissociation, producing ions that cannot cross the cell membrane (*13*). The accumulation of these ions is toxic, inhibiting metabolic reactions, causing membrane rupture, and disrupting intracellular pH homeostasis (*13*).

The potent antimicrobial activity of organic acids in their dissociated form (*13*) was evident in the acid treatments. Semi-preserved samples treated with CA, LA and AA showed reduced mesophilic counts and no significant changes in psychrotrophic counts over the 16 days of storage, with none reaching the 5-log limit. Therefore, the use of organic acids extended the shelf life of semi-preserved oysters by at least four days compared with the NaCl treatment and by at least 13 days compared with the water treatment.

### Sensory evaluation of marinated raw oyster meat

Sensory evaluation is a crucial indicator for assessing changes in seafood freshness during storage (*8*). The mean values and standard deviations for characteristic, spoiled, putrid and acidic odours are shown in Table 3. The analysis showed that all acid treatments differed significantly from the water treatment in terms of characteristic odour, with the AA treatment receiving the lowest scores for this parameter.

**Table 3:** Sensory parameters of oysters semi-preserved using different solutions and the evolution during storage at 4 °C for 16 days

*t*(storage)/ day	Treatment				
	W	NaCl	CA	LA	AA
Characteristic odour					
2	(5.4±2.0)^aeA^	(6.9±2.1)^afA^	(5.1±1.9)^beA^	(2.5±1.5)^bdfAd^	(1.0±0.8)^cdA^
7	(6.8±1.8)^aeAB^	(6.4±1.8)^afAB^	(4.5±2.0)^beAB^	(4.6±1.9)^bdfAB^	(3.3±1.5)^cdAB^
11	(7.2±2.1)^aeAB^	(5.6±2.0)^afAB^	(2.0±1.7)^beAB^	(1.9±1.8)^bdfAB^	(1.5±1.5)^cdAB^
16	(6.3±2.1)^aeB^	(2.5±2.3)^afB^	(3.6±1.1)^beB^	(3.1±1.5)^bdfB^	(0.8±0.6)^cdB^
Spoiled					
2	(0.0±0.0)^a^	(0.0±0.0)^aA^	(0.1±0.2)^aA^	(0.1±0.2)^aA^	(0.0±0.0)^aA^
7	(0.0±0.0)^aA^	(0.0±0.0)^aA^	(0.3±0.4)^aA^	(0.1±0.4)^aA^	(0.0±0.0)^aA^
11	(0.1±0.3)^aA^	(0.2±0.3)^aA^	(0.5±0.6)^aA^	(1.1±1.3)^aA^	(0.2±0.4)^aA^
16	(0.2±0.3)^aB^	(1.3±1.3)^aB^	(0.5±0.5)^aB^	(1.0±1.4)^aB^	(0.3±0.7)^aB^
Putrid					
2	(0.0±0.0)^aA^	(0.0±0.0)^aA^	(0.1±0.2)^aA^	(0.0±0.1)^aA^	(0.0±0.0)^aA^
7	(0.0±0.0)^aAC^	(0.0±0.0)^aAC^	(0.0±0.1)^aAC^	(0.2±0.4)^aAC^	(0.0±0.0)^aAC^
11	(0.1±0.2)^aAC^	(0.1±0.2)^aAC^	(0.1±0.2)^aAC^	(0.3±0.4)^aAC^	(0.1±0.3)^aAC^
16	(0.1±0.1)^aBC^	(0.2±0.3)^aBC^	(0.0±0.1)^aBC^	(0.2±0.3)^aBC^	(0.0±0.1)^aBC^
Acid					
2	(0.0±0.5)^bcA^	(0.3±0.2)^bcA^	(0.4±0.4)^bA^	(1.0±0.4)^bA^	(6.6±1.5)^aA^
7	(0.3±0.5)^bcA^	(0.1±0.2)^bcA^	(0.3±0.4)^bA^	(0.2±0.4)^bA^	(6.0±1.5)^aA^
11	(0.2±0.4)^bcA^	(0.6±1.0)^bcA^	(0.2±0.3)^bA^	(0.6±0.6)^bA^	(4.4±1.7)^aA^
16	(0.1±0.1)^bcA^	(0.0±0.0)^bcA^	(1.7±0.8)^bA^	(0.6±1.0)^bA^	(4.6±1.4)^aA^

Different lowercase letters in superscript within the same row indicate significant differences (p<0.05) among treatments. Different uppercase letters in superscript within the same column indicate significant differences (p<0.05) among storage periods. W=distilled water (control), NaCl=5 % sodium chloride (basic solution) and basic solution with the addition of 2 % acid: citric (CA), lactic (LA) and acetic (AA)

The intensity of spoiled odour, which is associated with lipid oxidation, tended to increase during storage. Among the acid treatments, only AA obtained spoiled odour scores similar to W, while LA and CA had higher scores. Despite these differences, overall scores remained low, with median values on day 16 of 0.45, 0.30 and 0.00 for LA, CA and AA, respectively.

Putrid odour scores remained low throughout the storage period, with no significant differences among treatments. Putrid odour is generally associated with high microbial counts and elevated TVB-N values; however, although water and NaCl treatments were unsuitable for human consumption after 16 days of storage, the intensity of putrid odour remained low. This may pose a potential consumer risk, as spoilage may not be readily perceived from the sensory characteristics of the product.

As expected, acid odour scores were higher in the acid treatments than in water and NaCl. However, LA and CA received overall low scores, with median values of 0.15 and 0.20, respectively, while AA obtained the highest scores, with a median of 5.35. Among the acid treatments, LA and CA showed overall sensory profiles more closely resembling those of fresh oysters.

Colour is one of the main quality attributes evaluated by consumers prior to purchase (*46*). The mean values and standard deviations of luminosity (L*), chromatic coordinates a* and b*, and total colour difference (Δ*E*) obtained from the instrumental colour analysis are shown in Table 4. L* values decreased significantly during storage, while no significant temporal variation was detected for a*. For b* and Δ*E*, significant interactions between storage time and treatments were observed. The LA treatment showed reduced b* values only after 16 days of storage. For Δ*E*, the water treatment resulted in increased values on day 7, while on day 16 the NaCl treatment showed higher values and the AA treatment showed lower values. In *C. gigas*, colour changes are associated with protein denaturation, which may explain the observed temporal variations (*47*).

**Table 4:** Colour parameters L*, a*, b* and total colour difference value Δ*E* for semi-preserved oysters with different treatments and stored at 4 °C for 16 days

*t*(storage)/ day	Treatment				
	W	NaCl	CA	LA	AA
a*					
0	4.79^A^	4.79^A^	4.79^A^	4.79^A^	4.79^A^
2	(3.3±0.6)^aA^	(4.3±0.8)^aA^	(6.3±2.0)^bA^	(6.1±1.8)^bA^	(4.1±0.5)^aA^
7	(3.9±1.2)^aA^	(3.8±0.7)^aA^	(6.3±0.3)^bA^	(6.7±1.2)^bA^	(4.6±1.4)^aA^
16	(1.7±0.5)^aA^	(3.3±0.1)^aA^	(6.4±1.2)^bA^	(6.3±1.8)^bA^	(4.3±1.1)^aA^
b*					
0	-14.99^aA^	-14.99^aA^	-14.99^aA^	-14.99^aB^	-14.99^aA^
2	(-14.5±1.8)^bA^	(-16.5±1.0)^bA^	(-13.6±2.1)^bA^	(-17.6±1.4)^b B^	(-14.7±1.2)^bA^
7	(-12.2±1.7)^cA^	(-15.3±0.7)^cA^	(-12.4±0.7)^cA^	(-15.0±2.4)^cB^	(-14.7±1.0)^cA^
16	(-13.7±1.5)^eA^	(-9.5±0.3)^eA^	(-12.3±1.8) ^deA^	(-10.6±0.5)^dA^	(-15.4±0.7)^eA^
L*					
0	65.19^C^	65.19^C^	65.19^C^	65.19^C^	65.19^C^
2	(68.6±4.7)^abA^	(64.9±2.0)^aA^	(71.2±1.6)^abA^	(68.0±0.4)^abA^	(72.3±2.5)^bA^
7	(76.1±1.7)^abB^	(66.5±4.1)^aB^	(72.9±1.7)^abB^	(70.5±5.3)^abB^	(77.1±5.3)^bB^
16	(61.8±1.5)^abC^	(65.±4.8)^aC^	(67.4±5.5)^abC^	(64.3±0.6)^abC^	(65.8±3.2)^bC^
Δ*E*					
0	-	-	-	-	-
2	(5.2±2.6)^abB^	(2.5±0.8)^bB^	(6.8±1.6)^aA^	(4.3±1.0)^abB^	(7.3±2.6)^aA^
7	(11.4±1.5)^aA^	(3.4±2.0)^bAB^	(8.4±1.3)^abA^	(7.4±3.5)^abB^	(12.0±5.3)^aAB^
16	(5.1±0.7)^aB^	(6.9±0.8)^a^	(6.1±0.7)^aA^	(5.0±0.4)^aB^	(2.9±0.6)^aB^

Lowercase letters in superscript in the same row indicate significant differences between mean values (p<0.05) for each treatment. Uppercase letters in superscript in the same column indicate significant differences between mean values (p<0.05) for each storage period. W=distilled water (control), NaCl=5 % sodium chloride (basic solution) and basic solution with addition of 2 % acid: citric (CA), lactic (LA) and acetic (AA)

Using the control (W) as the reference for treatment comparisons, it was found that for a*, only CA and LA were significantly higher. For b*, no significant differences were detected until day 7, while by day 16, LA showed significantly lower values. For L*, only AA differed significantly from W. In general, no significant differences among treatments were observed for Δ*E*, except for NaCl, which differed significantly from W on day 7.

## CONCLUSIONS

Marinating raw oysters in weak organic acids proved to be an effective strategy for controlling mesophilic and psychrotrophic bacterial growth, extending the shelf-life to at least 16 days under refrigerated storage at 4 °C. Among the tested acids, acetic acid showed the greatest positive effect on microbiological and physicochemical parameters, maintaining acceptable TVB-N and TBARS values and comparatively higher pH values than the other acids. However, it also resulted in the highest acid odour scores and the lowest characteristic oyster odour scores, which may limit its sensory acceptance. Lactic and citric acids, on the other hand, produced overall similar results to each other, with sensory attributes more closely resembling those of fresh oysters, higher characteristic oyster odour scores, and lower acid odour scores, although they had slightly higher spoiled odour scores, even though absolute values remained low. Therefore, while acetic acid appears to be the most effective for ensuring microbiological safety and shelf-life extension, lactic and citric acids may be preferable when prioritising sensory quality.

Future research should explore combinations of acids, particularly acetic with lactic or citric, to optimise both safety and sensory attributes. Studies should also investigate lower amounts of acetic acid to minimise acid odour without compromising antimicrobial efficacy, assess consumer acceptance through sensory panels, and evaluate the impact of marination on volatile compound profiles and bioactive components. Additionally, testing different oyster species and varying storage temperatures would further validate and expand the applicability of these findings.
